# Expression and gene regulatory network of SNHG1 in hepatocellular carcinoma

**DOI:** 10.1186/s12920-021-00878-2

**Published:** 2021-01-26

**Authors:** Chaoran Zheng, Shicheng Yu

**Affiliations:** 1 Key Laboratory of Combinatorial Biosynthesis and Drug Discovery, School of Pharmaceutical Sciences, Wuhan University, Wuhan, 430071 China; 2 Guangzhou Institutes of Biomedicine and Health, Chinese Academy of Sciences, 190 Kaiyuan Avenue, Guangzhou Science Park, Luogang District, Guangzhou, 510530 China; 3 Guangzhou Regenerative Medicine and Health Guangdong Laboratory, Bioland Laboratory, Guangzhou, 510005 China

**Keywords:** LIHC, HCC, TCGA, ceRNA, SNHG1, Survival

## Abstract

**Background:**

Small nucleolar RNA host gene 1 (SNHG1), a long noncoding RNA (lncRNA), is a transcript that negatively regulates tumour suppressor genes, such as p53. Abnormal SNHG1 expression is associated with cell proliferation and cancer. We used sequencing data downloaded from Genomic Data Commons to analyse the expression and interaction networks of SNHG1 in hepatocellular carcinoma (HCC).

**Methods:**

Expression was examined using the limma package of R and verified by Gene Expression Profiling Interactive Analysis. We also obtained miRNA expression data from StarBase to determine the lncRNA-miRNA-mRNA–related RNA regulatory network in HCC. Kaplan–Meier (KM) analysis was performed using the survival package of R. Gene Ontology annotation of genes was carried out using Metascape.

**Results:**

We found that SNHG1 was overexpressed and often amplified in HCC patients. In addition, SNHG1 upregulation was associated with the promotion of several primary biological functions, including cell proliferation, transcription and protein binding. Moreover, we found similar trends of small nucleolar RNA host gene 1 (SNHG1), *E2F8* (E2F transcription factor 8), *FANCE *(FA complementation group E) and *LMNB2 *(*encodes lamin B2*) expression. In the SNHG1-associated network, high expression levels of SNHG1 (log-rank P value = 0.0643), *E2F8* (log-rank P value = 0.000048), *FANCE* (log-rank P value = 0.00125) and *LMNB2* (log-rank P value = 0.0392) were significantly associated with poor survival. Single-cell analysis showed that *E2F8* may play an important role in tumorigenesis or cancer development.

**Conclusions:**

Our results highlight the benefit of utilizing multiple datasets to understand the functional potential regulatory networks of SNHG1 and the role of SNHG1 in tumours.

## Background

Liver cancer is the fourth leading cause of death from cancer in the world [1] and is one of the most fatal and algetic tumours. Hepatocellular carcinoma (HCC) is the most common histological type of primary liver cancer, occurring in 75–85% of patients with primary liver cancer, and Africa and Asia have the highest incidences [1, 2]. Recent research shows that HCC patients tend to have a family history of HCC and that the pathological mechanisms are complex [3, 4]. Overall, medical practice has changed rapidly in the last few years with the aid of rapidly expanding genome sequencing techniques [5], and precision medicine is gradually becoming an emerging field in the treatment and prevention of diseases. Although medical researchers have overcome the challenges of assessing large amounts of data, the application of genetic testing in cancer treatment is progressing slowly. A primary reason for this slow progress is that large amounts of accumulated patient data are independent and discrete. Thus, researchers need to determine relationships between diverse data and their correlations with disease.

Noncoding RNAs (ncRNAs) are a class of RNA molecules that do not participate in protein coding but do regulate gene expression, contributing to many physiological and pathological processes, including tumours [6]. Long noncoding RNAs (lncRNAs) are an important subcategory of ncRNAs with lengths longer than 200 nucleotides [7, 8]. Recent studies show that lncRNAs participate in various biological processes, such as cell differentiation [9], signal transduction [10] and posttranscriptional regulation [11], that are conducive to the progression of tumours and many other diseases [12]. Increasing evidence suggests that lncRNAs can function alone or in cooperation with microRNAs (miRNAs). Typically, lncRNAs act as ceRNAs to regulate messenger RNAs (mRNAs). This mode is known as the “lncRNA-miRNA-mRNA network”. This regulation has been reported in some tumour-related studies, but traditional research methods are insufficient for analysing multi-omics data [13, 14]. Nevertheless, ceRNA network analysis can shed light on ncRNA expression alterations in cancers and uncover relationships between altered RNAs.

To reveal the roles of the lncRNA-miRNA-mRNA network in human HCC, we sought to identify ceRNA networks by identifying similar regulatory networks from an extensive collection of data from Genomic Data Commons (GDC) using a stringent bioinformatics pipeline. The identified ceRNA networks may help in identifying molecular subtypes or new biological markers to classify HCC patients. Moreover, by examining differences in patient survival based on the expression of each RNA subtype, our ceRNA networks offer potential clinical utility for personalized treatments and therapeutic targets.

## Methods

### Data downloading and organization

The gdc-client method was used to download RNA-seq, miRNA and clinical data from the GDC database via TCGA-Assembler (http://www.compgenome.org/TCGA-Assembler/) of R (3.6.2) [15]. A total of 424 RNA-seq, 425 miRNA and 377 clinical data points were included in the study. Duplicates were filtered out before analysis of the metadata download from the GDC database. We also removed samples that were not from primary tumours or normal solid tissues. The TMM method implemented in edgeR of R (3.6.2) [16] was used to normalize RNA-seq and miRNA raw count data, after which we removed genes with a logcpm of expression below 1 in more than half of the samples. We used the limma [17] package in R (3.6.2) (https://www.r-project.org/) to screen genes and miRNAs showing significant differential expression between tumour and normal tissues. Significant differentially expressed genes (DEGs) (based on P < 0.01 and fold change (FC) > 2) were identified in the RNA-seq and miRNA expression profiles, and volcano plots and bar plots were constructed in R to visualize these DEGs. Expression analysis of different sample types (tumour vs. normal tissues) was performed with GEPIA (http://gepia.cancer-pku.cn/) [18]. Next, we assessed the levels of protein expression for these genes based on immunohistochemistry data obtained from the Human Protein Atlas database (https://www.proteinatlas.org/) [19–21].

### ceRNA network analysis

Competing lncRNA-mRNA pairs were identified using the gdcCEAnalysis function of GDCRNATools [15]. The precise identification of lncRNA-miRNA-mRNA interactions was based on three lines of evidence. First, the hypergeometry of shared miRNAs was evaluated to reduce the number of lncRNA-mRNA pairs; hypergeometric analysis was performed to determine whether a lncRNA-mRNA pair shares many miRNAs. Second, we excluded all lncRNA and mRNA pairs that exhibited variable correlations in expression. Finally, the lncRNA-mRNA pairs retained were required to have regulation similar to that of all shared miRNAs. After these steps, several lncRNA-miRNA-mRNA interactions were identified, and functional analysis was then carried out. To identify the number of miRNAs related to lncRNAs and mRNAs, miRNA-mRNA and miRNA-lncRNA interactions were obtained from StarBase v2.0 [22]. The network was visualized using Cytoscape (3.4.0) (https://cytoscape.org) [23, 24].

### Univariate survival analysis

GdcSurvival Analysis in GDCRNATools was employed to analyse the association with overall survival for each gene [15]. First, we divided the patients into high- and low-expression groups based on the median expression level. Then, Kaplan–Meier (KM) analysis was performed using the survival package, and the results were examined in univariate survival analysis. The final results are presented as the hazard ratio (HR) and significance (log-rank P value).

### Functional analysis

Gene Ontology (GO) annotation for the genes was carried out using the Metascape database (http://metascape.org/gp/index.html#/main/step1) [25]. Then, Online Mendelian Inheritance in Man (OMIM) (https://www.omim.org/) was applied to analyse associations between diseases and the genes in the lncRNA-miRNA-mRNA network.

### Single-cell analysis

To assess gene expression in different cell types, we obtained single-cell sequencing data from a previous report [26]. Gene expression was normalized by log2 (filteredexpr + 1) as mentioned in [26]. Cell clusters were generated with seurat software [27], and the cell clustering information was retrieved from the same report [26]. The expression of these genes in endothelial cells and macrophages was evaluated, and the results of gene expression in different cell clusters are illustrated in a violin plot.

## Results

### RNA expression in HCC

**Fig. 1:**
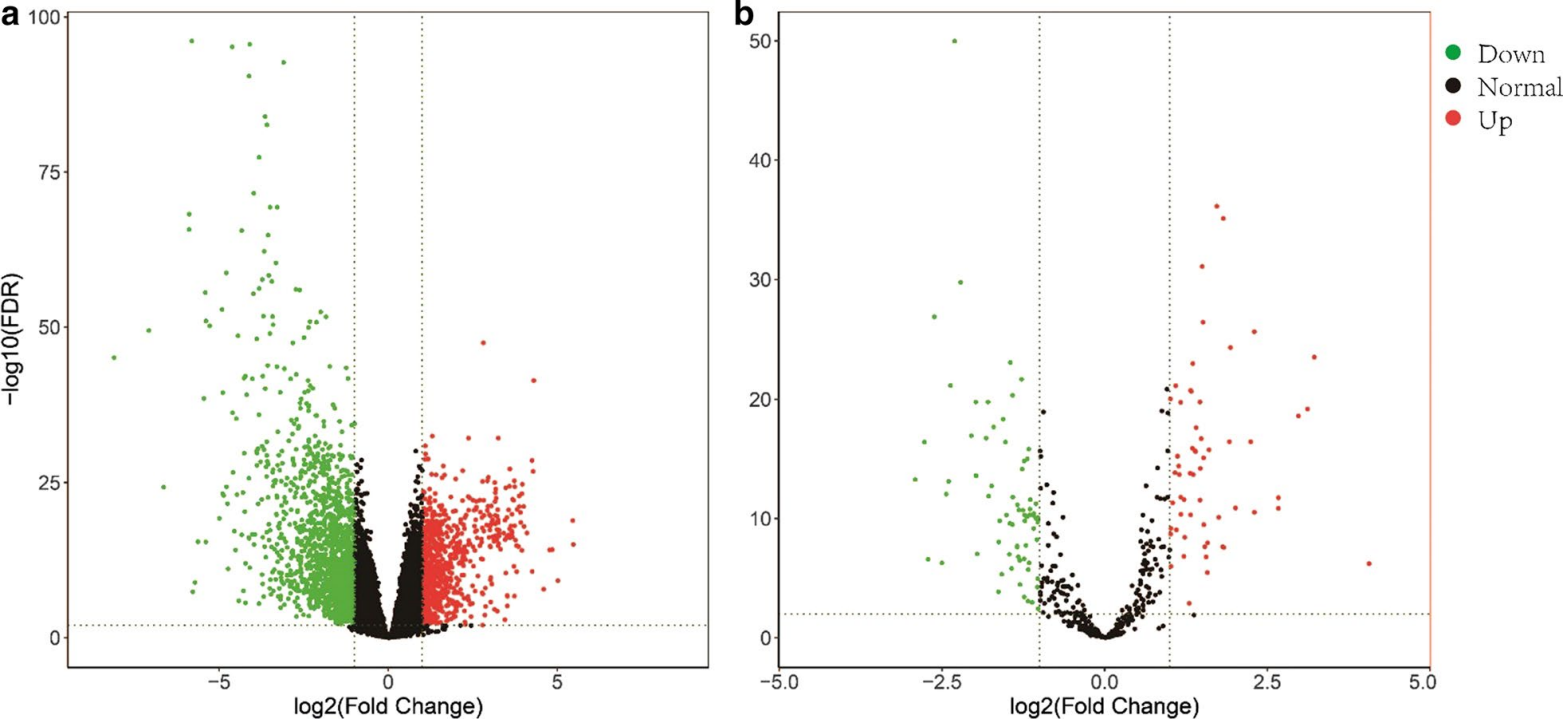
Genes differentially expressed in HCC. A volcano plot was constructed in R with the expression matrix of DEGs (**a**) and DEMs (**b**), showing − log10 (FDR) versus log2 (fold change)

Using the limma package, we identified 807 upregulated DEGs, 1590 downregulated DEGs, 59 upregulated differentially expressed miRNAs (DEMs), and 71 downregulated DEMs (Fig. 1). In total, we identified 2397 DEGs and 130 DEMs.

### Biological interaction networks in HCC

**Fig. 2:**
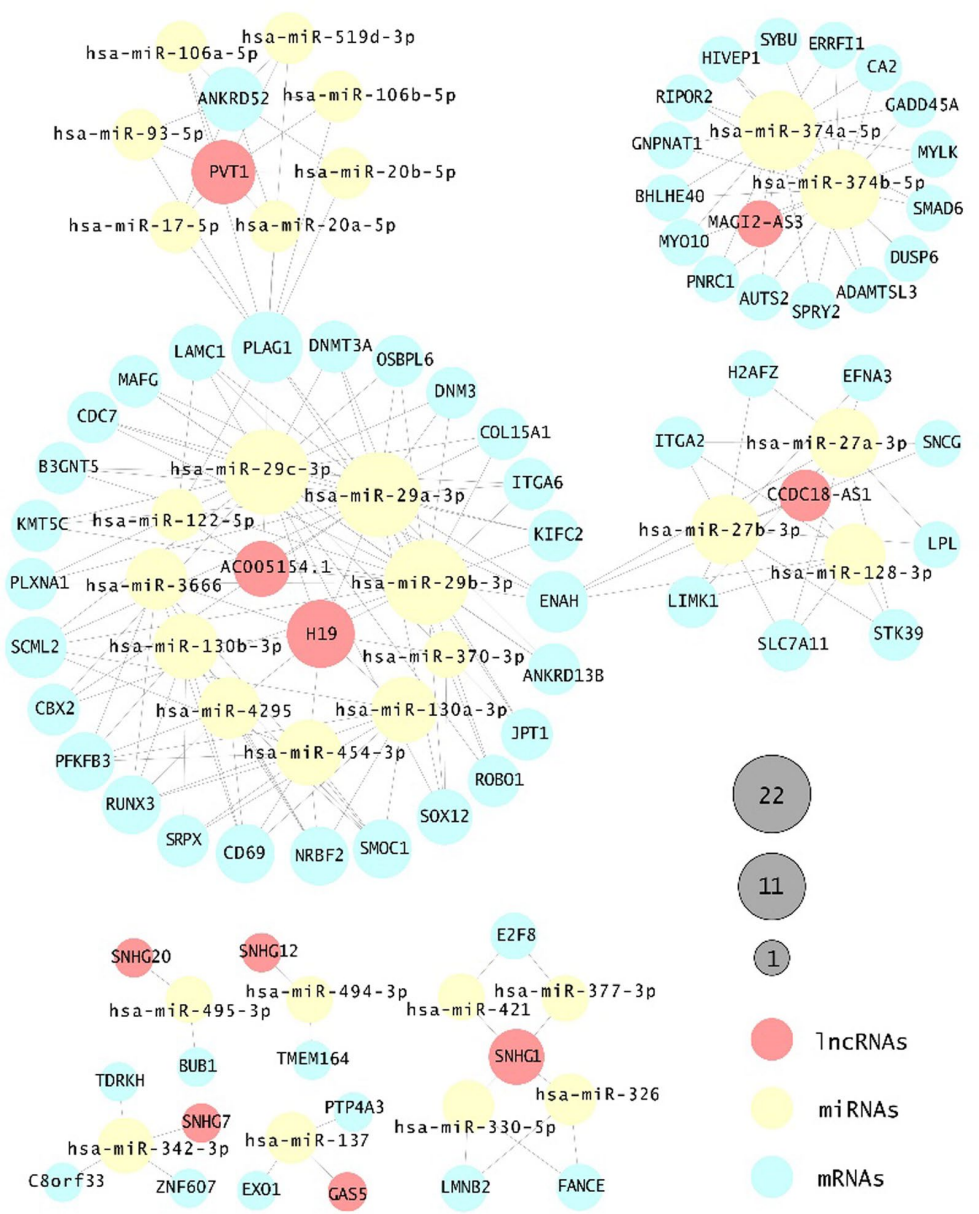
Visualization of the ceRNA network. The ceRNA network containing 101 nodes and 209 edges in HCC demonstrated paired bonds among lncRNA-miRNA-mRNA interactions. Red, yellow, and blue represent lncRNAs, miRNAs, and mRNAs, respectively. Circle size represents the number of edges connected to the node

We next sought to determine the biological interaction networks in HCC by using internally incorporated miRNA-mRNA and miRNA-lncRNA interactions, and we identified seven mRNA-miRNA-lncRNA networks (Fig. 2). All the ceRNA networks share similar regulation of all miRNAs interacting with the lncRNA-mRNA pairs. For example, the neighbouring genes of *MAGI2-AS3*, namely, *ADAMTSL3*, *AUTS2*, *BHLHE40*, *DUSP6*, *ERRFI1*, *GADD45A*, *GNPNAT1*, *HIVEP1*, *MYLK*, *MYO10*, *PNRC1*, *RIPOR2*, *SMAD6*, *SPRY2*, and *SYBU,* share the same miRNAs. Additionally, two miRNAs (hsa-miR-374a-5p and hsa-miR-374b-5p) mediated the *MAGI2-AS3*-mRNA network.

### Univariate survival analysis of coexpressed genes in HCC

Abnormal expression of many genes affects the duration of human survival. By constructing a ceRNA network, we identified hub genes with which miRNAs might interact. In addition, we established survival-related networks. Then, we focused on SNHG1 and related genes that predict the same direction of correlation with prognosis.

**Fig. 3:**
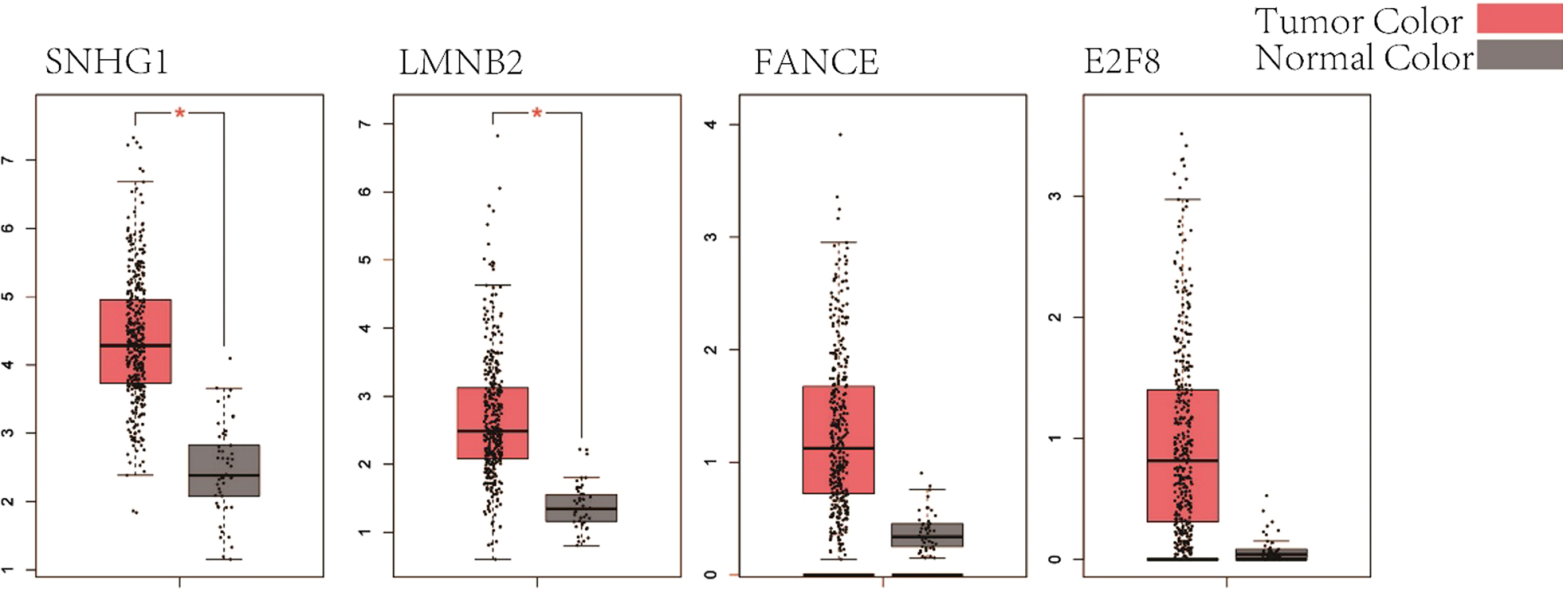
Difference expression analysis of SNHG1*, LMNB2, FANCE,* and *E2F8* by GEPIA. Boxplots depicting expression levels in HCC versus those in normal solid tissue

**Fig. 4:**
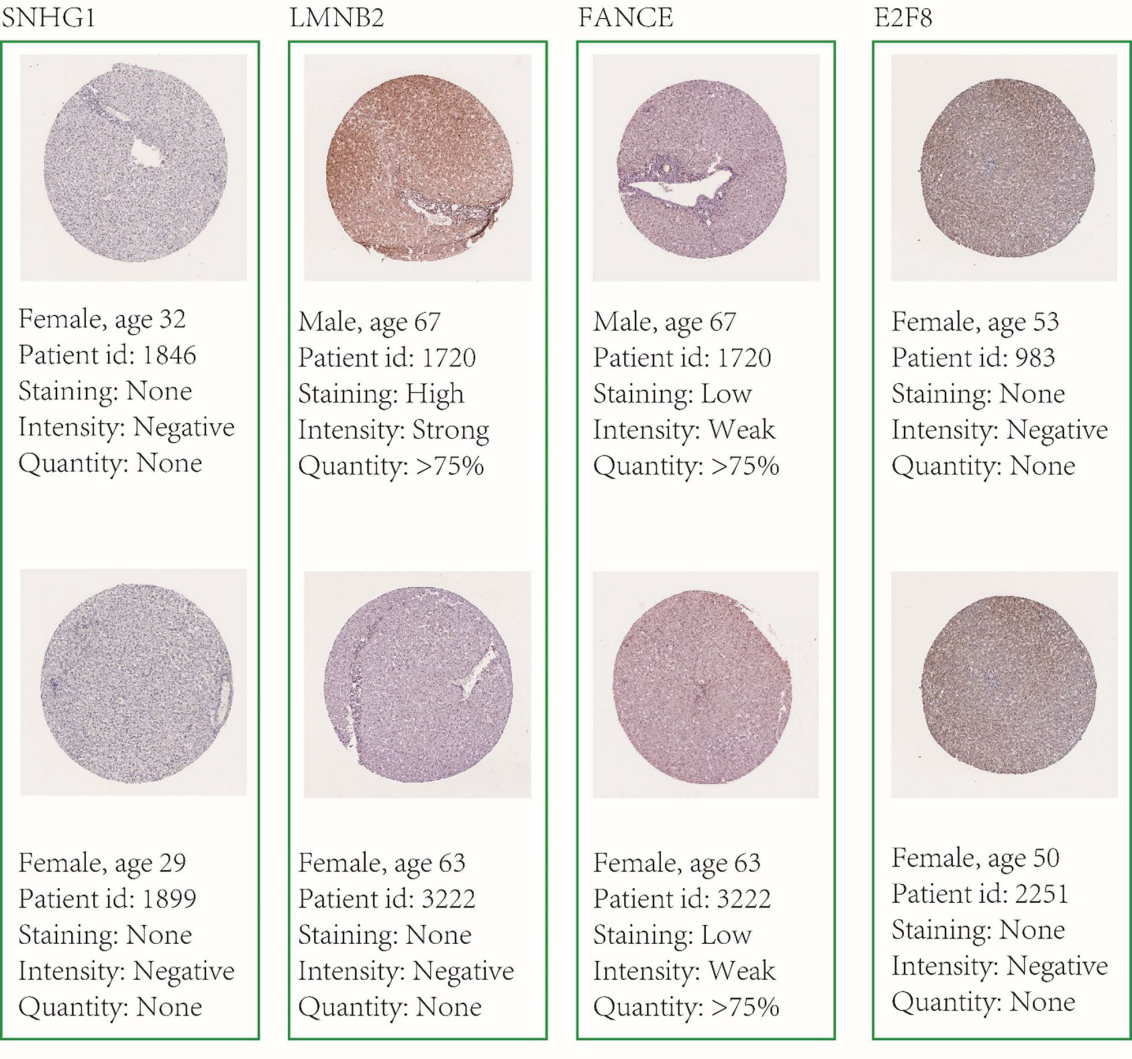
Translational levels of SNHG1*, LMNB2, FANCE,* and *E2F8* in Normal tissue. The immunohistochemistry result was obtained from Human Protein Atlas database. Number of tumour is 369; and number of normal is 50

We evaluated SNHG1 transcription levels in HCC studies from GDC. Data in the GDC database revealed significantly higher mRNA expression of SNHG1 in HCC samples than in normal solid tissue samples (log-rank P value = 6.43E-18) (Table 1), with a fold difference above 2. The results of the expression analysis for SNHG1*, LMNB2, FANCE,* and *E2F8* were verified by GEPIA (Fig. 3). We also verified the low protein levels of these four genes in normal samples using the Human Protein Atlas database (Fig. 4). Four miRNAs (hsa-miR-421, hsa-miR-377-3p, hsa-miR-330-5p, and hsa-miR-326) mediated the SNHG1 network. Thus, SNHG1 expression may serve as a prognostic indicator in HCC.

**Table 1:** Biological functions of the genes in the SNHG1-related network

Gene symbol	Description	OMIM	Type	LogFC	P value
SNHG1	Small nucleolar RNA host gene 1	603222	Long non coding	1.495397836	6.43E−18
*LMNB2*	E2F transcription factor 8	612047	Protein coding	1.115366226	5.95E−13
*FANCE*	FA complementation group E	613976	Protein coding	1.459672439	9.12E−14
*E2F8*	Lamin B2	150341	Protein coding	3.729047657	8.89E−27

The table lists 5 genes in the SNHG1-related network and the following information: Gene symbol, Description, OMIM, Type, LogFC (trimmed binary logarithm of average expression level fold change), and P value

**Fig. 5:**
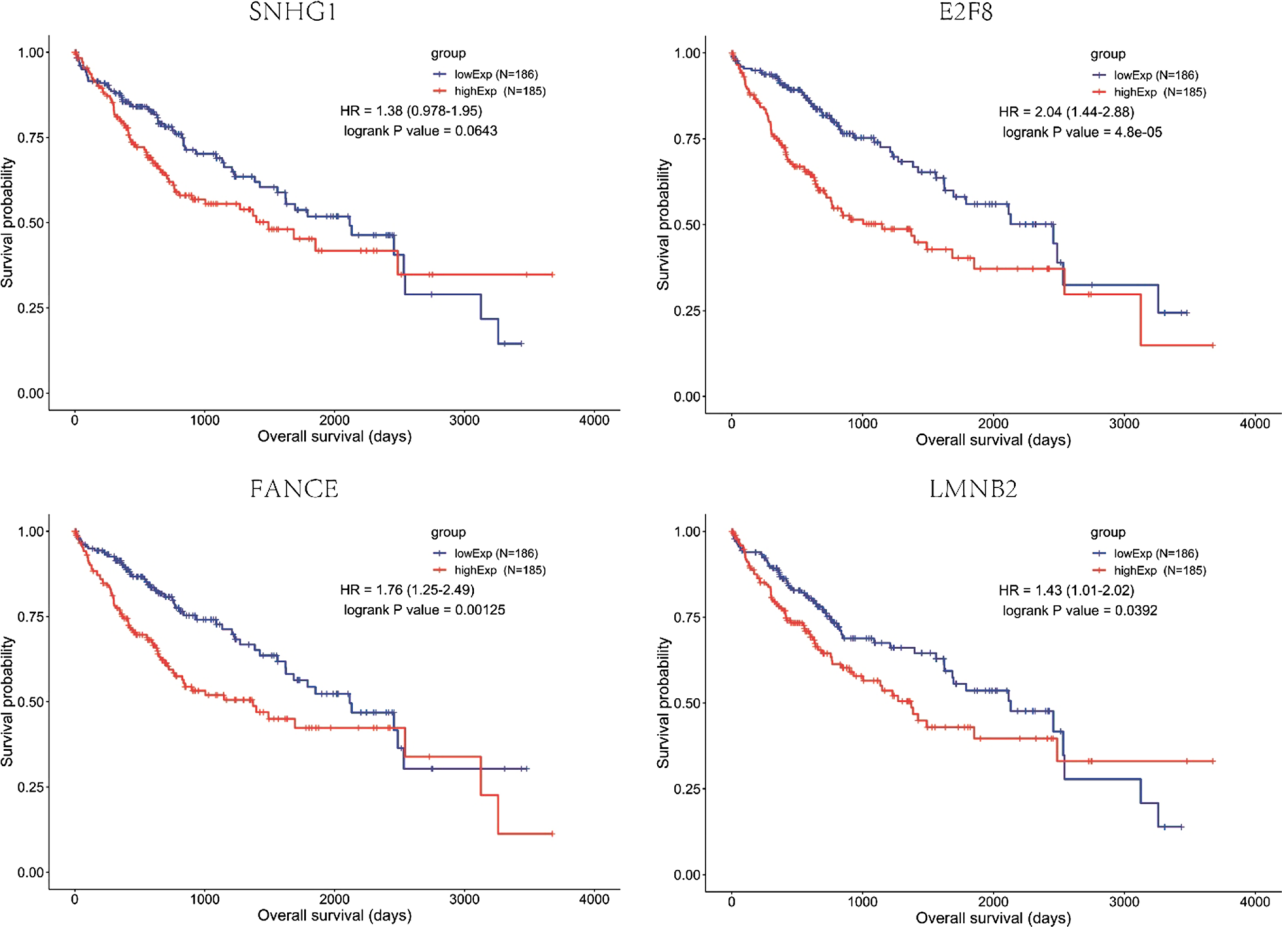
Univariate survival analysis. Analysis of prognostic RNAs screened from the ceRNA network. Overall survival analysis of SNHG1, *E2F8*, *FANCE* and *LMNB2* in HCC

To better understand the functions of the coexpressed genes (*E2F8*, *FANCE* and *LMNB2*) correlated with SNHG1, we compared survival time among patients with different levels of gene expression and found that high expression of SNHG1 was associated with decreased survival (log-rank P value = 0.0643) (Fig. 5). Additionally, in the SNHG1-associated network, high expression levels of *E2F8* (log-rank P value = 0.000048), *FANCE* (log-rank P value = 0.00125) and *LMNB2* (log-rank P value = 0.0392) correlated significantly with poor survival (Fig. 5); these genes affect transcription activity, DNA repair, and protein binding, respectively. Overall, we detected interaction effects of *SNHG1* and the other three genes on survival.

### Functional analysis of the coexpressed genes correlated with SNHG1 in HCC

To examine the roles that the 3 coexpressed genes we identified play in the cell, we annotated all three genes and SNHG1 using the Custom Analysis function of Metascape (http://metascape.org/) (Table 1). All of these genes are involved in primary life processes, such as transcription activity, DNA repair, and protein binding. Two of these genes encode plasma proteins, and *E2F8* encodes a transcription factor with a winged helix-turn-helix DNA-binding domain.

### SNHG1-related mRNA expression in endothelial and macrophage populations

**Fig. 6:**
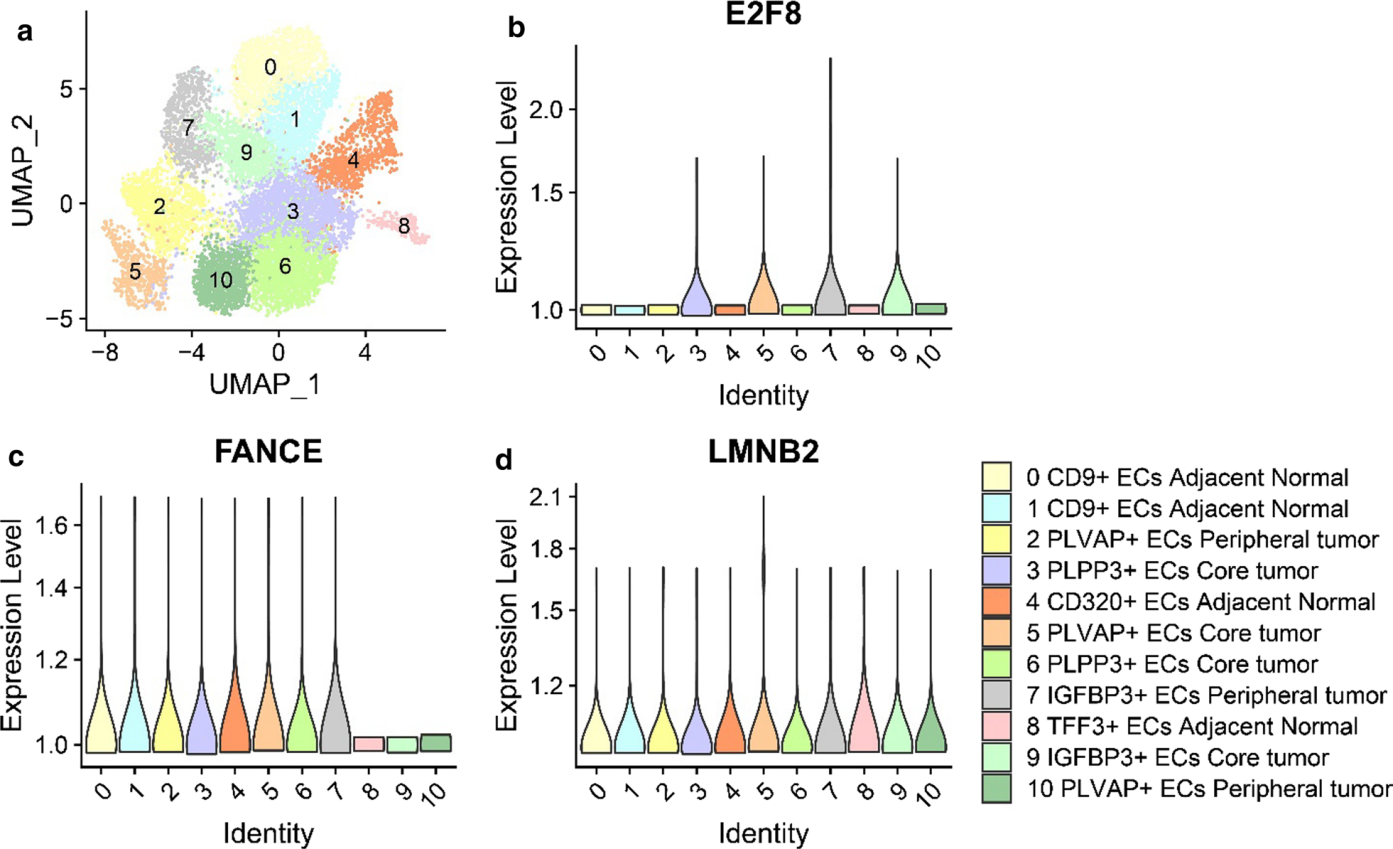
Tumor-Associated Endothelial Cells. **a** UMAP analysis and graph-based clustering results were obtained from [26]. Identifies 11 endothelial cell clusters from 14 HCC patients and one healthy donor in liver. **b**–**d** Violin graph represent gene expression in endothelial cell population. ECs: endothelial cells

**Fig. 7:**
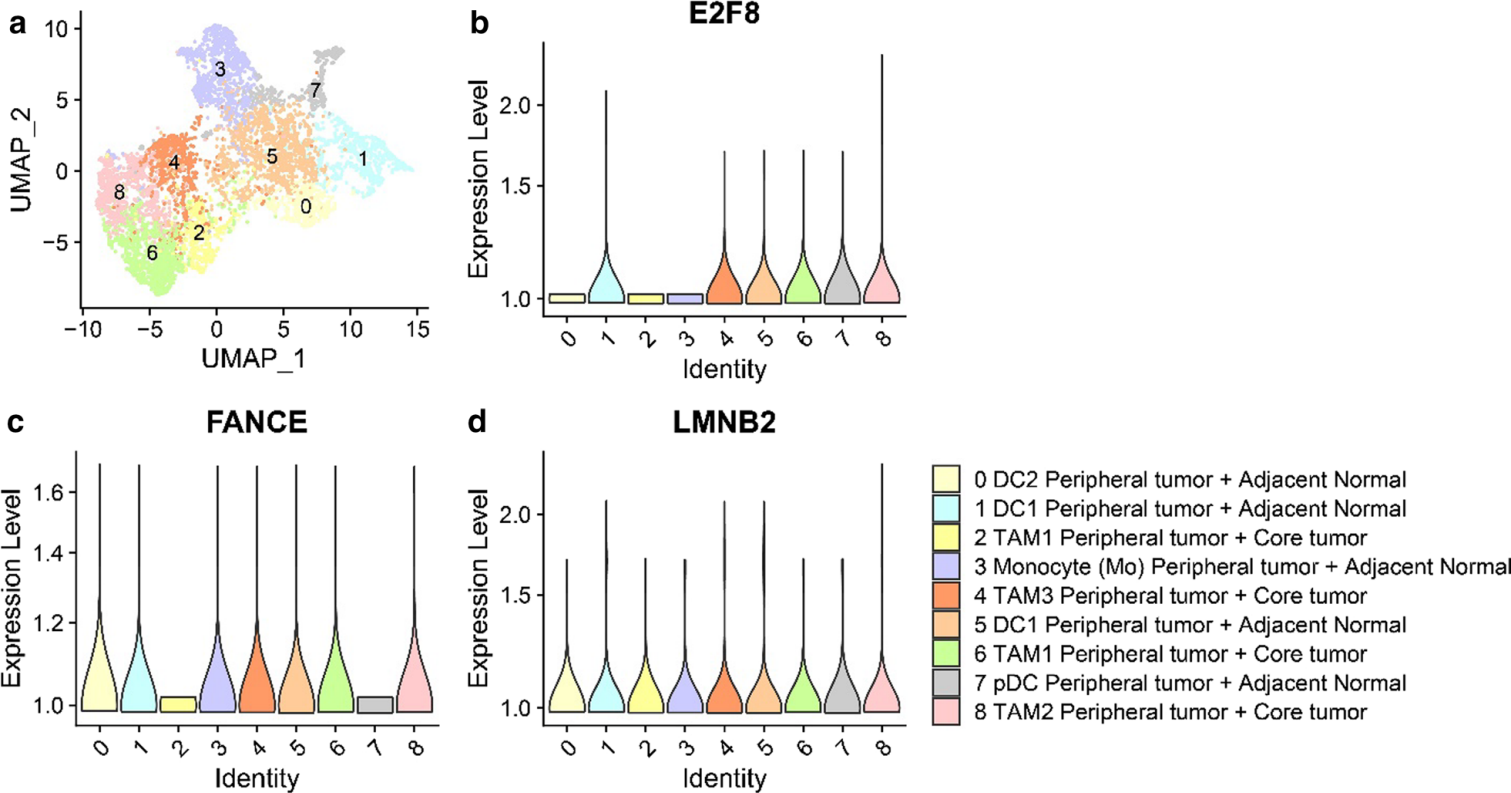
Tumor-Associated Myeloid Cells **a** UMAP analysis and graph-based clustering results were obtained from [26]. Identifies 9 Myeloid cell clusters from 14 HCC patients and one healthy donor in liver. **b**–**d** Violin graph represent gene expression in Myeloid cell population. DC1, DC2, pDC: dendritic cells; TAMs: tumor-associated macrophages

We analysed the expression of the three coexpressed genes described above in endothelial cells (ECs) and macrophages, which were significantly different based on single-cell data [26] (Figs. 6 and 7). *LMNB2* was found to be uniformly expressed in the 11 EC clusters. Specifically, *E2F8* was expressed at low levels in clusters 0, 1, 4, and 8, corresponding to normal cells [26]. At the same time, *FANCE* was expressed at low levels in clusters 8 (adjacent normal), 9 (core tumour), and 10 (peripheral tumour), indicating no difference between tumour and normal cells. Mononuclear phagocyte-based subclustering analysis also suggested low levels of *E2F8* expression in peripheral tumours and adjacent normal tissues. However, *LMNB2* and *FANCE* were uniformly expressed in the 9 macrophage clusters. These results suggest that several clusters of *E2F8*-deficient endothelial cells and macrophages may be tuned to regulate cancer processes in HCC.

## Discussion

Using RNA-seq, miRNA and clinical data downloaded from the GDC database, we identified 807 and 1590 upregulated and downregulated DEGs and 59 and 71 upregulated and downregulated DEMs, respectively. A total of 2397 DEGs and 130 DEMs were found, and by constructing a ceRNA network, we detected hub genes with which miRNAs may interact. We observed 7 lncRNA-miRNA-mRNA interactions and performed survival analysis on all genes in the networks.

We analysed data for 377 patients from the GDC database and found that high SNHG1 expression indicated poor survival; high *E2F8, FANCE* and *LMNB2* levels were also associated with poor patient survival. A previous study revealed a strong negative correlation between SNHG1 expression and survival time in patients with high-risk neuroblastoma (NB) [28]. In addition, downregulation of *E2f8* resulted in a series of embryonic defects affecting vasculature and cell survival [29]. *FANCE* is reported to be a vital factor influencing the poor survival of FANCE-deficient cells among breast cancer cell lines [30], and upregulation of *LMNB2* is associated with significantly poorer prognosis in lung adenocarcinoma patients [31]*.* Therefore, SNHG1 may affect survival through *E2F8, FANCE* and *LMNB2*. Further experiments are needed to examine interactions in this SNHG1-related network.

CeRNAs are RNA molecules that affect mRNA expression by competing for miRNAs in the same interaction network. Disruption of interactive networks can cause disease, including various cancers [32]. It is widely recognized that SNHG1 plays roles in cell proliferation and tumours. The patients showed poor prognosis when SNHG1 was upregulated, which indicated the prognostic value of SNHG1 [33, 34]. They also identified several miRNA-SNHG1 subnetworks (such as hsa-miR-195-5p-SNHG1, hsa-miR-497-5p-SNHG1, and hsa-miR-101-3p-SNHG1) and several mRNA-SNHG1 pairs (such as *MYLK-AS1*-SNHG1, *CHEK1*-SNHG1, and *KIF2C*-SNHG1) in the ceRNA regulatory network of HCC [33, 34]. Through ceRNA network studies, SNHG1 was found to be important in HCC (SNHG1-hsa-miR-101-3p-*KIF2C*) [33], colorectal cancer (SNHG1-hsa-miR-484-*ORC6*, SNHG1-has-miR-423-5p-*EZH2*, and SNHG1-let-7b-5p-*ATP6V1F*) [35], lung adenocarcinoma (SNHG1-hsa-miR-21-5p-*RALGPS2*, SNHG1-hsa-miR-326-*RALGPS2*, SNHG1-hsa-miR-377-3p-*RALGPS2*) [36], nasopharyngeal carcinoma (SNHG1-miR-145-5p-*NUAK1*) [37] and prostate cancer (SNHG1-miR-199a-3p-*CDK7*) [38]. SNHG1 was also found to be highly expressed in posterior longitudinal ligament patients [39], cholangiocarcinoma [40], and osteosarcoma [41, 42]. In the ceRNA network established in this study, we found SNHG1-hsa-miR-421-*E2F8*, SNHG1-hsa-miR-377-3p-*E2F8*, SNHG1-hsa-miR-330-5p-*LMNB2*, and SNHG1-hsa-miR-326-*FANCE* axes in HCC. *E2F8* was found to be required for cell cycle progression and proliferation [43], *FANCE* to be essential for protection against chromosome breakage [44], and *LMNB2* to be required for early replication and transcription [45]. These genes mediate links between miRNAs, which affect the development of HCC as a network. However, studies with large samples are needed to confirm how these miRNAs are connected.

SNHG1 is a lncRNA that is generally thought to be produced by a small nuclear RNA host gene. Cell proliferation and changes in the tumour protein p53 occur when SNHG1 is abnormally expressed [46]. Considering the very important functions of SNHG1, its 3 coexpressed genes can be expected to be related to disease, with altered expression in many human diseases. Of these genes, *E2F8* is usually accompanied by overexpression of genes during progression of the cell cycle and cell proliferation [47]. Expression of *E2F* target genes is required in the S phase of the cell cycle. As a part of this nuclear complex, FANCE interacts with BRCA1 at sites of DNA damage by activating the downstream protein FANCD2 [44]. *LMNB2*, replicated in the first minute of S phase in humans, is essential for early replication and transcription [48]. In addition, LMNB2, which is located on the inner side of the nuclear envelope, is required for nuclear stability, chromatin structure, and gene expression [48].

To explore the cell type specifically of these genes in tumour and normal samples, we analysed their expression in endothelial cells and macrophages and found low expression of *E2F8* in normal epithelial cells, which may indicate that its presence promotes cancer. This is also consistent with the conclusion drawn from TCGA data, whereby no difference between *LMNB2* and *FANCE* was detected in these two cell types from normal and tumour samples.

## Conclusions

In this work, we identified 7 lncRNA-miRNA-mRNA interactions. Furthermore, we demonstrated that *ADAMTSL3*, *AUTS2*, *BHLHE40*, *DUSP6*, *ERRFI1*, *GADD45A*, *GNPNAT1*, *HIVEP1*, MYLK, *MYO10*, *PNRC1*, *RIPOR2*, *SMAD6*, *SPRY2*, and *SYBU* show the same expression trends as SNHG1 in HCC. By generating lncRNA-miRNA-mRNA interaction networks and performing univariate survival analysis of the genes coexpressed with lncRNAs in HCC, we demonstrated that SNHG1 has the same effect on patient survival as *E2F8*, *FANCE* and *LMNB2.* Single-cell analysis also demonstrated that *E2F8* may play an important role in tumorigenesis or cancer development. Therefore, SNHG1, *E2F8*, *FANCE* and *LMNB2* may represent therapeutic targets for HCC with complex regulation. Taken together, our research provides genetic resources for capturing the majority of essential genes involved, which will yield a global view of the minimum set of genes and pathways. The biological interaction network of SNHG1-related lncRNA-miRNA-mRNA interactions provides insight into the function of SNHG1 and its molecular regulation in HCC.

## Abbreviations

HCC: Hepatocellular carcinoma
GEPIA: Gene Expression Profiling Interactive Analysis
LIHC: Liver hepatocellular carcinoma
SNHG1: Small nucleolar RNA host gene 1
MiRNA: MicroRNA
LncRNA: Long noncoding RNA
ceRNAs: Competing endogenous RNAs
DEGs: Differentially expressed genes
DEMs: Differentially expressed miRNAs
GO: Gene Ontology
PPI: Protein–protein interaction
TCGA: The Cancer Genome Atlas
E2F8: E2F transcription factor 8
FANCE: FA complementation group E
LMNB2: Lamin B2

## Availability of data and material

The RNA-seq, miRNA and clinical data of LIHC (Liver hepatocellular carcinoma) were downloaded from the DATA PORTAL of GDC (Genomic Data Commons) https://portal.gdc.cancer.gov/projects/TCGA-LIHC. Immunohistochemistry data were downloaded from the Human Protein Atlas database (https://www.proteinatlas.org/) under gene symbols described in Table 1. Single-cell sequencing data is available from NCBI Gene Expression Omnibus (GEO: GSE156337).
